# Influence of lip closure on alveolar cleft width in patients with cleft lip and palate

**DOI:** 10.1186/1746-160X-7-3

**Published:** 2011-01-26

**Authors:** Wolfgang Eichhorn, Marco Blessmann, Oliver Vorwig, Gerd Gehrke, Rainer Schmelzle, Max Heiland

**Affiliations:** 1 Department of Oral and Maxillofacial Surgery, General Hospital Balingen, Tübinger Str. 30, 72336 Balingen, Germany; 2 Department of Oral and Maxillofacial Surgery, University Medical Center Hamburg-Eppendorf, Martinistr. 52, 20246 Hamburg, Germany; 3 Department of Oral and Maxillofacial Surgery, Henriettenstiftung Hannover, Marienstr. 72-90, 30171 Hannover, Germany

## Abstract

**Background:**

The influence of surgery on growth and stability after treatment in patients with cleft lip and palate are topics still under discussion. The aim of the present study was to investigate the influence of early lip closure on the width of the alveolar cleft using dental casts.

**Methods:**

A total of 44 clefts were investigated using plaster casts, 30 unilateral and 7 bilateral clefts. All infants received a passive molding plate a few days after birth. The age at the time of closure of the lip was 2.1 month in average (range 1-6 months). Plaster casts were obtained at the following stages: shortly after birth, prior to lip closure, prior to soft palate closure. We determined the width of the alveolar cleft before lip closure and prior to soft palate closure measuring the alveolar cleft width from the most lateral point of the premaxilla/anterior segment to the most medial point of the smaller segment.

**Results:**

After lip closure 15 clefts presented with a width of 0 mm, meaning that the mucosa of the segments was almost touching one another. 19 clefts showed a width of up to 2 mm and 10 clefts were still over 2 mm wide. This means a reduction of 0% in 5 clefts, of 1-50% in 6 clefts, of 51-99% in 19 clefts, and of 100% in 14 clefts.

**Conclusions:**

Early lip closure reduces alveolar cleft width. In most cases our aim of a remaining cleft width of 2 mm or less can be achieved. These are promising conditions for primary alveolar bone grafting to restore the dental bony arch.

## Background

The treatment of children with a cleft lip and palate remains a challenge. Beginning at birth, it is necessary to balance several aspects of treatment such as growth, esthetics, function, and psychosocial development. Especially in children with a complete bilateral cleft lip and palate, many problems remain unsolved. Apart from intrinsic tissue deficiency and anatomic aberrations, there is difficulty in restoring the orbicularis oris muscle, in creating a philtrum, and in lengthening the columella. Furthermore, benefit of early orthopedic treatment is still questioned. Unrestricted premaxillary growth also gives rise to many problems. Surgeons have not reached consensus regarding best type and timing of lip- and palatal closure. Similarly, orthodontists have not reached agreement on early management of the alveolar segment position before lip closure. Some promoted the use of active or passive intra-oral appliances in order to normalize alveolar segment position before lip closure. This would enable the surgeon to operate with less tension on the soft tissues. Others have advocated the use of extra-oral strapping placed. Finally, the influence of surgery on further growth and stability after treatment are topics still under discussion [1-10]. According to the Muenster treatment protocol an early lip closure is performed at the age of 4-6 months [7], while Anastassov and Joos prefer an age of 3 months [5]. According to the Hamburg treatment protocol lip closure is performed even earlier at the age of 8 weeks in the mean and can be classified as a very early lip closure.

The aim of the present study was to investigate the influence of early lip closure on the width of the alveolar cleft using dental casts.

## Methods

37 patients (21 male, 16 female) were evaluated in this study. A total of 44 clefts were investigated using plaster casts, 30 unilateral and 7 bilateral clefts. All infants received a passive molding plate a few days after birth. The age at the time of closure of the lip was 2.1 month in average (range 1-6 months). The one patient with the age of 6 months suffered from a hydrocephalus, whereby an earlier operation could not be realized. The surgical procedure was performed according to Tennisson or Millard. The closure of the soft palate was performed at the age of 8.5 months in average (range 4-17 months).

Orthodontic plaster casts were obtained at the following stages: shortly after birth, prior to lip closure, and prior to soft palate closure. We determined the width of the alveolar cleft before lip closure and prior to soft palate closure measuring the alveolar cleft width from the most lateral point of the premaxilla/anterior segment to the most medial point of the smaller segment according to Sillmann and Robertson et al. [1,11].

Due to the small number of cases, a descriptive analysis was performed. The reduction of alveolar cleft width after lip closure in mm and in percent to the original alveolar cleft width were calculated.

## Results

After lip closure 15 clefts presented with a width of 0 mm, meaning that the mucosa of the segments of both sides were in direct contact. 19 clefts showed a width of up to 2 mm and 10 clefts were still over 2 mm wide. The relative reduction compared to the original cleft width revealed a reduction of 0 percent in 5 five clefts. In one case an original cleft width of 0 mm before lip closure did not enhance and stayed small. Small clefts stayed small. 6 clefts showed a reduction of 1-50%, 19 a reduction of 51-99% and 14 a reduction of 100%. A 100% reduction means that the cleft segments were in direct contact (table 1).

**Table 1:** Alveolar cleft width and reduction of cleft width at time of soft palate closure

**Alveolar cleft width (mm)**	**Number of patients**
0	15
up to 2	19
more than 2	10
**Reduction of cleft width (%)**	
0	5
1-50	6
51-99	19
100	14

**Figure 1:**
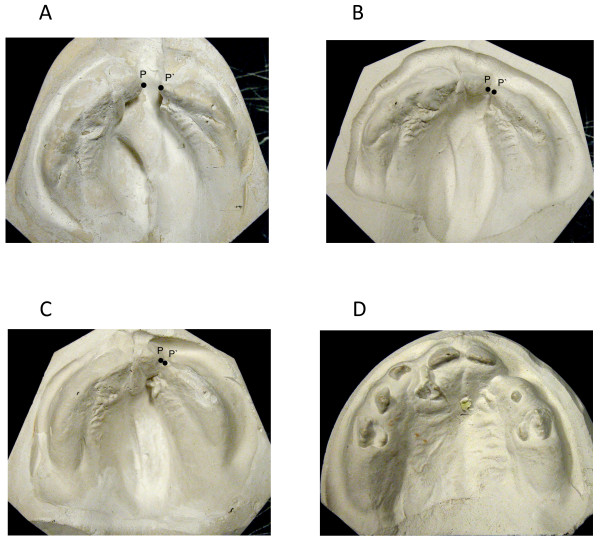
**Patient with cleft lip and palate**. P and P' denote the medial and the lateral edge of the cleft on a continuation of the line marking the crest of the ridge. a. 6 days old, 6 mm alveolar cleft width b. before lip closure at the age of 1 months, after treatment with feeding plate c. before closure of soft palate at the age of 7 months, 1 mm alveolar cleft width d. before alveolar bone graft at the age of 20 months

**Figure 2:**
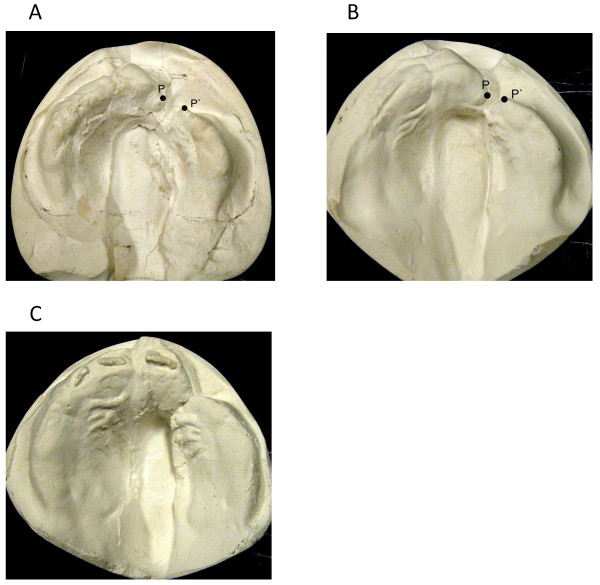
**Patient with left clip and palate**. P and P' denote the medial and the lateral edge of the cleft on a continuation of the line marking the crest of the ridge. a. 1 day old, 4 mm alveolar cleft width b. before lip closure at the age of 2 months, after treatment with feeding plate c. before closure of soft palate at the age of 10 months, soft tissues in contact

Additional dental casts obtained in single cases at later surgical procedures prove a rapid initial reduction of alveolar cleft width followed by a reduced velocity of movement (Figure 1, 2).

## Discussion

Active maxillary appliances are constructed to move alveolar segments in a predetermined manner with controlled force. The use of pin-retained appliances to expand collapsed alveolar segments while retracting the premaxilla in the case of a bilateral cleft and in unilateral cleft cases has been repeatedly advised [12-16]. In contrast, passive maxillary appliances do not provide any force. They act as a fulcrum for the forces created by the surgical lip closure, to contour and mold the alveolar segments in a predictable fashion [6,17]. Also a nasoalveolar molding as soon as possible after birth and nonsurgical lip adhesion by placing a tape across the upper lip have been proposed. The tape aids in the closure of the cleft, decreases the width of the base of the nose and helps to approximate the lip [6,17-20]. In our study a passive feeding plate in combination with an early lip closure with a restored orbicularis oris muscle was used for molding with 34 clefts being less than 2 mm wide at the time of soft palate closure. Therefore, an active appliance and two-staged lip repair as advocated seemed not to be necessary [21]. This achieved approximation would facilitate primary bone grafting of the alveolar cleft, if desired. The use of calvarian bone instead of bone from the iliac crest or mandible seems to be a promising alternative in bridging narrow alveolar defects [22].

In the 7^th ^week of pregnancy a cleft lip and palate inhibit the closure of the muscle rings of the mimic musculature and on the pharynx. The facial midsagittal axis is deviated to the non-cleft side because the muscles of the midface and lip are not attached to the septo-vomerine growth center. These insufficient muscular stimuli lead to skeletal changes we observe in cases of a cleft lip. Via reconstruction of the musculature the bones, for the most part, are able to develop normally. Keeping this in mind corrective surgery should be carried out as early as possible at the age of 3-4 months without using preoperative orthodontic appliances [5,7,23-27]. In our study, lip closure was performed even a little bit earlier (average 2.1 months) and passive feeding plates to facilitate breast feeding and to prevent tongue displacement in the palatal gap were used.

After lip closure the intercanine width, the growth of the arch depth and the intercanine width were significantly reduced showing an immediate effect of lip closure on maxillary arch shape. In the period between lip closure and palatal closure growth of the palatal arches changed into direction of the non-cleft controls while growth velocity of the intercanine width and the anterior arch remained less than the non-cleft controls [4,15,28].

As a modern alternative for analyzing orthodontic plaster cast models a 3D digital stererophotogrammetry can be used. This may also help to facilitate the documentation [29].

Attempts to close the lip cleft in-utero in a lamb model proved to have the advantage of scarless wound healing in the fetus and would also have positive effects on the alveolar cleft width. There was no inhibition of maxillary growth in the animals that underwent in-utero cleft lip repair in contrast to the neonatal group showing significant maxillary retrusion. However both lip repairs, the in-utero and neonatal group, produced significant shorter lips than the contralateral noncleft sides requiring a secondary lip revision. Thereby, the purpose of an intrauterine repair is defeated today [30-32].

## Conclusions

Early lip closure reduces alveolar cleft width. In most cases our aim of a remaining cleft width of 2 mm or less can be achieved. These are good conditions for primary alveolar bone grafting to restore the dental bony arch.

## Competing interests

The authors declare that they have no competing interests.

## Authors' contributions

WE and MH conceptualized the paper. WE, MH, MB, OV drafted and edited the manuscript. GG and RS were responsible for the treatment algorithm and performed the surgical procedures. All authors have read and approved the final manuscript.
